# Metabolic Profiling of Patients with Schizophrenia

**DOI:** 10.1371/journal.pmed.0030363

**Published:** 2006-08-22

**Authors:** Rima Kaddurah-Daouk

## Abstract

Kaddurah-Daouk discusses a new study that found that patients with schizophrenia have an abnormal cerebrospinal fluid composition. In half of the patients, the composition returned to normal with antipsychotic therapy.

Metabolomics (also referred to as metabonomics) is the comprehensive study of the metabolome, the repertoire of biochemicals (or small molecules) present in cells, tissue, and body fluids. The study of metabolism at the global or “-omics” level is a new but rapidly growing field that has the potential to have an impact upon medical practice [1–4]. At the center of metabolomics is the concept that an individual's metabolic state is a close representation of the individual's overall health status. This metabolic state reflects what has been encoded by the genome and modified by environmental factors.

Today, clinicians capture only a very small part of the information contained in the metabolome, as revealed by a defined set of blood chemistry analyses to define health and disease states. Examples include measuring glucose to monitor diabetes and measuring cholesterol for cardiovascular health. Such a narrow chemical analysis could potentially be replaced in the future with a metabolic signature that captures global biochemical changes in disease and upon treatment.

Such metabolic signatures could provide: (1) prognostic, diagnostic, and surrogate markers for a disease state; (2) the ability to subclassify disease; (3) biomarkers for drug response phenotypes (pharmacometabolomics); and (4) information about mechanisms of disease. Indeed, sophisticated metabolomic analytical platforms and informatics tools have recently been developed that make it possible to define initial metabolic signatures for several diseases [5–12].

## Biochemical Changes in Schizophrenia

A new study in *PLoS Medicine* by Elaine Holmes and colleagues is an early metabolomics experiment that attempts to identify biomarkers to assist in the diagnosis and treatment of schizophrenia [13]. The authors explore the use of a nuclear magnetic resonance–based metabolic profiling platform (metabonomics) to define biochemical changes in the cerebrospinal fluid (CSF) and biomarkers in first onset, drug-naïve patients with schizophrenia, and upon treatment with antipsychotic medications.

There are no validated biomarkers of schizophrenia that establish diagnosis or reliably predict response to treatment.

Schizophrenia is a devastating mental illness that is poorly understood at the molecular level [14]. There are no validated biomarkers of schizophrenia that establish diagnosis or reliably predict response to treatment. While antipsychotic therapies were proven effective in short-term trials, most patients discontinue treatment over time for lack of effectiveness or development of side effects, and not all patients respond similarly to these medications [15]. An important clinical feature of schizophrenia is that patients have a greater risk than the general population for developing obesity and metabolic disorders such as type 2 diabetes mellitus and the metabolic syndrome [16].

Holmes and colleagues found an abnormal biochemical profile in patients with schizophrenia or brief psychotic disorder when compared with healthy controls. The loading coefficients indicated that glucose, acetate, alanine, and glutamine resonances were predominantly responsible for separation of disease and control populations. Upon treatment with antipsychotic medications, this aberrant metabolic signature reverted to normal in half of the patients in their study. Their data suggest that early treatment of a first psychotic episode seems to help revert abnormal metabolic states to normal.

While these preliminary data are derived from only a small patient population (54 patients with schizophrenia or brief psychotic disorder) using one metabolomics platform that captures only a subset of the metabolome, the study nevertheless exemplifies the potential for metabolomics in the study of human disease. Impairments in glucose and acetate point to pathways of energy metabolism and lipid biosynthesis as possibly being impaired in schizophrenia. Further longitudinal studies, including randomization to treatment with larger patient populations, are needed to define effects of each drug on metabolic signatures. When defining disease signatures and biomarkers for schizophrenia, patients and controls must be very carefully matched; possible confounding effects of other medications and disease states have to be considered, as these could contribute to signatures and biomarker development. Replication and validation studies are needed in independent sets of patients and controls.

## Future Metabolomics Studies

Since no one metabolomics platform can capture the entire metabolome, the use of complementary metabolomics platforms could better define impaired pathways and highlight biomarkers. For example, by using a targeted lipidomics platform that reports back on more than 300 lipids, our group was able to highlight global lipid perturbations associated with schizophrenia and the use of three antipsychotic medications (unpublished data). Ongoing studies with an electrochemistry-based metabolomics platform are starting to define global changes in neurotransmitter pathways in schizophrenia.

Collectively, these metabolomics tools could provide valuable information about disease pathogenesis and result in metabolic signatures that could be developed as biomarkers for disease and progression. Comparative studies in CSF and plasma could help map central and peripheral changes in metabolism in schizophrenia and enable a more accessible way for biomarker development.

The observation that not all patients correct their aberrant metabolic profiles upon treatment with antipsychotics suggests that metabolic profiling could be used as an additional tool to complement clinical evaluation in defining drug response phenotypes and variation in response to therapy. Pharmacometabolomics is emerging as a new field that could complement pharmacogenomics by providing precise intermediate phenotypes for drug response. Metabolomics could add significantly to our understanding of both pharmacokinetic and pharmacodynamic properties of drugs. While in Holmes and colleagues' study drug effects were classified based on whether the antipsychotic was atypical or typical, a more precise mapping of the effect of each antipsychotic alone is warranted. Different atypical antipsychotics might have different mechanisms of action and solicit different responses in different patients.

Metabolomics has the potential to map early biochemical changes in disease and hence provide an opportunity to develop predictive biomarkers that can trigger earlier interventions. The authors suggest that early treatment seems to correct more effectively for biochemical changes noted in schizophrenia, allowing reversal to a more normal metabolic state.

## Conclusion

Holmes and colleagues' study illustrates the promise of metabolomics for the study of human disease. Metabolomics builds on more than a hundred years of findings in biochemistry and takes one of the oldest sciences to an “-omics” level. Data from metabolomics, when combined with other omics data, could enable a more global systems approach to the study of human disease.

## Abbreviations

CSF: cerebrospinal fluid
